# Relationship between self-management of patients with anxiety disorders and their anxiety level and quality of life: A cross-sectional study

**DOI:** 10.1371/journal.pone.0284121

**Published:** 2023-05-12

**Authors:** Xinyu Cao, Mei Feng, Ruyu Ge, Yan Wen, Jing Yang, Xiaolin Li

**Affiliations:** 1 Department of Pulmonary and Critical Care Medicine, West China Hospital / West China School of Nursing, Sichuan University, Chengdu, China; 2 West China Hospital / West China School of Nursing, Sichuan University, Chengdu, China; Chulabhorn Royal Academy, THAILAND

## Abstract

**Purpose:**

To explore the correlation between self-management and anxiety and quality of life in patients with anxiety disorders and to understand whether self-management can influence the relationship between anxiety and quality of life and provide a basis for improving their anxiety and quality of life.

**Methods:**

A cross-sectional survey of 245 patients with anxiety disorders in West China Hospital of Sichuan University was conducted using the Self-Management of Anxiety Disorders Scale, Self-rating anxiety scale (SAS), and World Health Organization Quality of Life-BBREF. The data were then analyzed using descriptive tests and Pearson correlation in SPSS 25. Bootstrap mediated effects tests were used to test the effect relationship between anxiety, quality of life and self-management scores in patients with anxiety disorders and Amos was used to establish the structural equation model.

**Results:**

The results of the correlation analysis showed that the total self-management score was positively correlated with the total quality of life score (r = 0.695, P <0.001), physical domain score (r = 0.552, P <0.001), psychological domain score (r = 0.661, P <0.001), social relations domain score (r = 0.542, P <0.001), and environmental domain score (r = 0.614, P <0.001). Additionally, it was negatively correlated with SAS score (r = –0.470, P <0.001). Self-management partially mediates the relationship between anxiety and quality of life, and the direct effect value of anxiety on quality of life was –0.44. The indirect effect value of self-management was –0.27, accounting for 38% of the total effect value.

**Conclusion:**

Self-management of patients with anxiety disorders was negatively correlated with anxiety and positively correlated with quality of life. It played a partially mediating effect between anxiety and quality of life. We recommend that healthcare providers pay attention to self-management interventions for patients with anxiety disorders to further improve their quality of life.

## Introduction

Anxiety disorders, also known as anxiety disorders, are a general term for a group of psychiatric disorders in which pathological anxiety symptoms are the main clinical stage, and are one of the most common clinical psychiatric disorders [1]; they are a major cause of disability. Approximately one-in-four individuals may suffer or have previously suffered from anxiety disorders [2,3]. Lifetime prevalence ranges from 4.8% in China to 31% in the United States [2]. An epidemiological study of anxiety disorders conducted in China in 2019 revealed that the prevalence of anxiety disorders in China amounted to 7.6%, with a 12-month prevalence of 5.0% [3].

Due to the characteristics of anxiety disorders, patients may suffer from comorbid emotional and behavioral conditions, such as depression, alcohol and drug abuse, and suicidal thoughts [2]. Currently, globally, the quality of life of individuals with mental disorders is poor, and public mental health has gradually become a hotspot of social concern [4–6]. Furthermore, the high disability rate of anxiety disorders, long-term medication, and young age of onset may lead to a reduced quality of life among patients with anxiety disorders [7]. In China, symptoms of anxiety and depression may significantly affect the quality of life of patients [8]. In a systematic evaluation, the quality of life of patients with anxiety disorders before and after treatment was found to be lower than that of the healthy population [9].

As a chronic psychiatric disorder, anxiety disorders do not only have a high incidence but also have poor treatment outcomes [3,10]. Sole reliance on treatment during hospitalization is inadequate and needs to be combined with self-management at home to achieve anxiety control and reduce the number of relapses [11,12]. In the psychiatric context, self-management, comprising emotional management and medication management, refers to all actions taken by patients on a daily basis to manage their psychiatric symptoms, avoid relapse, and improve their sense of well-being [13].

Self-management in other chronic diseases, such as cardiovascular diseases and endocrine diseases, has been extensively studied [14–17]. Study has shown that self-management is associated with disease symptoms, and that effective self-management can reduce disease symptoms [18]. In a study on chronic obstructive pulmonary disease (COPD), Bringsvor et al [18] found that a higher symptom burden resulted in a lower self-management score. However, relevant research on self-management for people with anxiety disorders is relatively scarce, [19,20] especially in China. Furthermore, due to the special cultural background of China, attention to patients with anxiety disorders has usually been inadequate, and few studies have assessed strategies to improve self-management at home. This cross-sectional survey is part of a larger intervention study that aims to improve patients’ ability to self-manage at home. This study initially examines the relationship between self-management and anxiety and quality of life when patients with anxiety disorders are treated at home. In addition, the study seeks to understand whether self-management improves the quality of life of patients with anxiety disorders, providing a new basis for future improvements in quality of life and the need for self-management interventions for patients with anxiety disorders.

## Methods

### Study design

A cross-sectional design was used for this study; this study is part of a larger interventional study. In a previous study, we developed a self-management scale for patients with anxiety disorders using the Delphi method [21]. We followed up that study with this study by investigating the correlation between self-management and anxiety and quality of life.

### Study population and sample

Patients with anxiety disorders followed up in the outpatient clinic of a mental health center from April 2021 to August 2021 were recruited using a convenience sampling method. Due to the COVID 2019 epidemic, the questionnaire was collected online. We have placed recruitment posters related to the study in the Mental Health Centre and interested participants can contact the staff via WeChat. The questionnaire was created as a link that could be filled in online through the Questionnaire Star software and sent to participants who met the study’s inclusion criteria via WeChat. After submitting the questionnaire, we were able to see the participant’s questionnaire information in the backend of the questionnaire. Inclusion criteria for patients with anxiety disorders to participate in the study were as follows: (a) a diagnosis of anxiety by a psychiatrist according to the International Classification of Diseases, 10^th^ Revision (ICD-10); (b) Chinese residency; and (c) ability to understand the questionnaires. Participants were excluded from this study if they (a) were diagnosed with other severe mental illnesses (bipolar disorder or schizophrenia); or (b) diagnosed with other chronic physical diseases (hypertension or diabetes mellitus). Out of 431 eligible patients, 245 patients completed the questionnaires, with a response rate of 56.84%.

### Data collection tools

#### Patient characteristics

Patient characteristics included age, sex, marital status, ethnicity, education level, monthly household income, work status, insurance, geographic area of housing, family history of psychiatric disorders and duration.

#### Self-rating anxiety scale (SAS)

The 20-item SAS was used to evaluate anxiety symptoms [18]. SAS is widely used and has good accuracy in evaluating psychometric properties in the Chinese population. Each item was scored on a 4-point Likert scale, ranging from 1 (never or rarely) to 4 (usually or always). Scores for the 20 items were summed to give an original score, which was then multiplied by 1.25 to give a total SAS score, ranging from 25 to 100. Anxiety was classified as none (25–49 points), mild (50–59 points), moderate (60–69 points), and severe (70–100 points). The scale has been tested as a reliable and valid tool for self-assessing anxiety levels: the Cronbach’s alpha coefficient for the scale was 0.82 [22]. In this study, the Cronbach’s α value was 0.88.

#### Self-management scale for patients with anxiety disorders

The Self-Management Scale for Patients with Anxiety Disorders contains two subscales: Disease Medical Management and Psychosocial Management subscales [20]. The Disease Medical Management subscale includes three factors of medication management, emotion management, and symptom knowledge; and the Psychosocial Management subscale includes three factors of self-efficacy, social functioning, and resource management. The scale has 31 items on a 5-point Likert scale, ranging from 0 to 4. Higher scores on this scale represent better self-management. This scale has good reliability and validity in measuring self-management in patients with anxiety disorders, the Cronbach’s alpha coefficient for the total scale was 0.920 [20]. In this study, Cronbach’s α was determined to be 0.94.

#### World Health Organization Quality of Life-BREF (WHOQOL-BREF)

This scale was developed under the auspices of the World Health Organization’s Quality of Life Group and revised by Fang Jiqian in Chinese. It is used to measure an individual’s subjective perception of overall quality of life and health, and has been classified as a health industry standard by the Chinese government [23,24]. This scale includes four domains of physical, psychological, social relationships, and environmental domains. It has 26 entries, and is scored from 1 (very poor/very unsatisfactory) to 5 (very good/very satisfactory). Each domain score is the average score for that domain multiplied by a factor of 4; the total score is the sum of the four domain scores. Higher scores represent better quality of life. This scale has been shown to have good reliability and validity: physical health (Cronbach’s a = 0.82), psychological health (Cronbach’s a = 0.81), social relationships (Cronbach’s a = 0.68), and environment (Cronbach’s a = 0.80) [23,25,26]. In this study, Cronbach’s α was 0.94.

### Data analysis

Data were analyzed using IBM SPSS software (version 25.0). Data were summarized as frequencies (n) and percentages (%). Kruskal-Wallis analysis was used to derive a normal distribution of self-management scores. Pearson’s correlation was used to analyze the correlation between self-management and anxiety and quality of life. P-value <0.05 indicated statistical significance. Bootstrapping was used to test the mediating effect of self-management on the relationship between anxiety and quality of life. A mediating effect path analysis chart was produced using Amos25.0 modeling and analysis.

### Ethical considerations

All procedures of this study were in accordance with the ethical standards of the institutional research committee and the Helsinki Declaration. This study has been approved by the ethics committee of XXXX (Approval number: 2019–961). Patients’ consent was also obtained at the time of completing the questionnaire. Prior to the start of the study, participants’ diagnoses were checked and verified, those who met the requirements for a diagnosis of anxiety disorder were given informed consent, informed of the purpose of the study and the tasks to be completed in the study, and confirmed that they fully understood the study and responded "I fully understand the study and volunteer to participate". In addition, the first part of the questionnaire was informed consent, where patients were asked to read the informed consent form and answer the questions "Do you wish to participate in this study" and "Do you wish your findings to be used for academic research". Selecting ’No’ to either of these two questions will automatically close the questionnaire and end the response.

## Results

### General characteristics of the patients (N = 245)

In this study, 75 (30.61%) participants were men and 170 (69.39%) participants were women. The age range was 16–71 years; the median age was 33 (27, 45) years. Table 1 shows the characteristics of enrolled participants. Eighty (32.65%), 144 (58.78%), and 21 (8.57%) participants were single, married, and widowed or divorced, respectively.

**Table 1:** Characteristics of study participants (N = 245).

Characteristics	N	%
**Age (M)**	33(27,45)	
**Sex**		
Men	75	30.61
Women	170	69.39
**Marital status**		
Unmarried	80	32.65
Married	144	58.78
Divorced/widowed	21	8.57
**Ethnicity**		
Han	238	97.14
Others	7	2.86
**Educational Level**		
Primary school	27	11.02
High school/technical college	94	38.37
University	102	41.63
Postgraduate	22	8.98
Monthly household income (CNY*)		
≤5000	61	24.90
5000–10000	78	31.84
>10000	106	43.27
**Work status**		
Student	30	12.24
Employed	146	59.59
Unemployed or retired	69	28.16
**Insurance**		
Yes	230	93.88
No	15	6.12
**Residential setting**		
Rural	78	31.84
Urban	167	68.16
**Family history of psychiatric disorders**		
Yes	66	26.94
No	179	73.06
**Duration of anxiety symptoms (years)**		
1	78	31.84
1~3	65	26.53
>3	102	41.63

Abbreviations: CNY, Chinese Yuan.

### Self-management scale, SAS, and WHOQOL-BREF scale scores

Data from the self-management scale, SAS, and quality of life scale were normally distributed; they were expressed as mean ± standard deviation (Mean ± SD) (Table 2).

**Table 2:** Scores of Self-management Scale, SAS, and WHOQOL-BREF scale.

Variables	Mean ± SD	Min	Max
**Self-management**	78.70 ± 22.66	11.00	124.00
**Disease medical**	39.36 ± 11.37	1.00	60.00
Medication management	15.18±5.71	0.00	20.00
Emotional management	8.35±3.32	0.00	12.00
Symptom knowledge	15.82±6.07	1.00	28.00
**Psychosocial**	39.34 ± 13.02	6.00	64.00
Social functioning	11.22±3.65	0.00	16.00
Resource management	12.54±5.80	0.00	24.00
Self-efficacy	15.58±5.92	0.00	24.00
**SAS**	52.23 ± 12.35	26.25	86.25
None	40.11±0.61	26.25	48.75
Mild	54.25±0.33	50.00	58.75
Moderate	64.04±0.43	60.00	68.75
Severe	75.07±1.09	70.00	86.25
**WHOQOL-BREF**	48.38 ± 10.65	19.52	72.96
Physiological	12.07 ± 2.81	4.00	20.00
Psychological	11.53 ± 3.33	4.00	19.32
Social	11.93 ± 3.28	4.00	20.00
Environmental	12.84 ± 3.05	4.00	20.00

Abbreviations: SAS, Self-Rating Anxiety Scale.

WHOQOL-BREF:World Health Organization Quality of Life-BREF.

### Correlation analysis of self-management with anxiety and quality of life

The results of the correlation analysis showed that the total self-management score had a positive correlation with total quality of life score (r = 0.695, P <0.001), physical domain score (r = 0.552, P <0.001), psychological domain score (r = 0.661, P <0.001), social relations domain score (r = 0.542, P <0.001), and environmental domain score (r = 0.614, P <0.001). Additionally, it was negatively correlated with SAS score (r = –0.470, P <0.001). Scores on the medical management of illness and psychosocial dimensions of self-management were positively correlated with scores on the physical, psychological, social and environmental dimensions of quality of life, but negatively correlated with scores on the SAS (Table 3).

**Table 3:** Correlation between Self-management and anxiety and quality of life.

	Quality of life	Physiological	Psychological	Social	Environmental	SAS score
Disease medical	0.551**	.408**	.534**	.428**	.504**	-.402**
	<0.001	<0.001	<0.001	<0.001	<0.001	<0.001
Psychosocial	0.729**	.604**	.684**	.570**	.628**	-.468**
	<0.001	<0.001	<0.001	<0.001	<0.001	<0.001
Self-management	0.695**	.552**	.661**	.542**	.614**	-.470**
	<0.001	<0.001	<0.001	<0.001	<0.001	<0.001

Note:

** indicates significant correlation at the 0.01 level (two-tailed).

### Path analysis of the mediating of self-management between anxiety and quality of life

**Fig 1:**
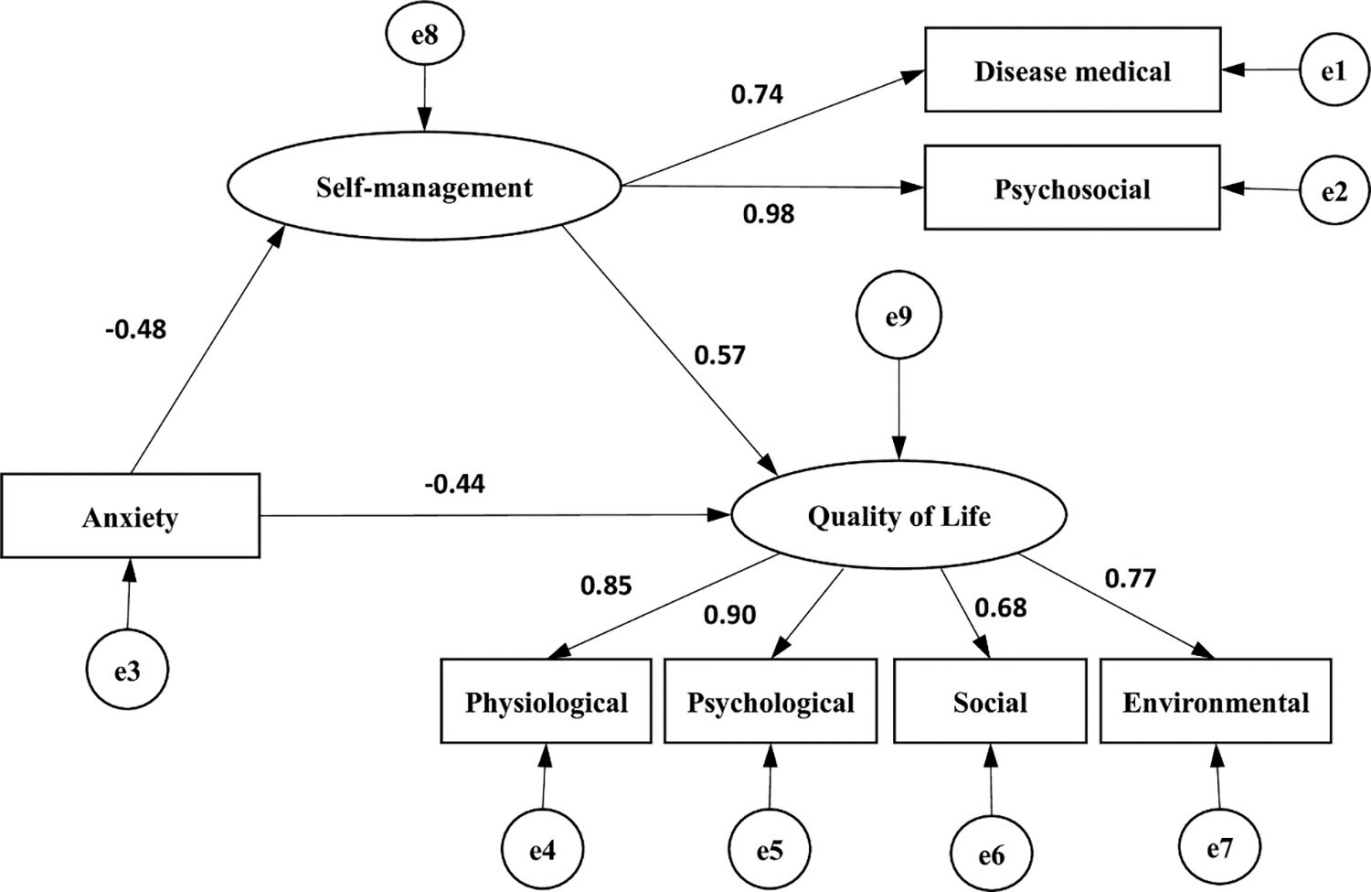
The mediating effect path diagram showing the mediating variable self-management.

The AMOS was introduced with self-management as the mediating variable, anxiety as the independent variable and quality of life as the dependent variable to model the outcome equation. The results showed that the path coefficients of the model were statistically significant (p < 0.05). The fitting indexes of the structural equation model were as follows: CMIN/DF = 3.213, RMSEA = 0.095, GFI = 0.957, NFI = 0.964, CFI = 0.975, and TLI = 0.956. Anxiety has a negative impact on quality of life, self-management has a positively impact on quality of life and anxiety negatively impacts on self-management. According to the path analysis diagram, the mediating effect was a*b = -0.27, the direct effect of anxiety on quality of life was -0.44 and the total effect was -0.71, with the mediating effect accounting for 38% of the total effect, as shown in Table 4 and Fig 1.

**Table 4:** Analysis of the mediating effect of self-management between anxiety and quality of life.

Intermediary Model	Total effect	Direct effect	Indirect effect
Anxiety-Self-Management-Quality of Life	–0.71	–0.44	–0.27

## Discussion

### Current status of self-management in patients with anxiety disorders

The self-management score in this study was slightly higher than that reported by Morita et al. [27] The disparity may be explained by the fact that the study population of Morita et al [27] included not only patients with anxiety disorders but also patients with bipolar disorder and depression. However, it is worth noting that in this study, the lowest score on the medical management of illness dimension of self-management was 1, indicating that some patients had a significant lack of knowledge related to anxiety symptom recognition, medication management and emotional management. This reminds healthcare professionals that they should increase education on disease related knowledge for anxious patients, such as through some online live video courses, graphic knowledge push or offline health talks and discharge education, so as to improve patients’ disease knowledge and promote self-management [28–30].

### Current quality of life of patients with anxiety disorders

In terms of quality of life, the lowest scores for physical, psychological and social and environmental aspects for people with anxiety disorders were all 4, meaning that they were very dissatisfied with these aspects, which also indicates that people with anxiety disorders do not have a high quality of life. The results of this study are similar to those of previous studies, in that patients with anxiety disorders have a generally lower quality of life [31–33]. These findings may be due to the fact that anxiety disorders may cause a range of comorbidities, such as somatic symptoms and psychological stress, which can lead to a lower quality of life. Higgins et al [34] confirmed the idea that the quality of life of patients with anxiety disorders decreases as the duration of symptoms increases [34].

### Analysis of correlation between self-management and anxiety and survival quality

Self-management was negatively associated with SAS, indicating that the more severe the patient’s anxiety symptoms, the worse the self-management behavior. Some studies on other diseases found that disease symptom burden affects self-management behaviors. Bringsvo et al [18] showed that the higher the symptom burden, the poorer the self-management of patients with COPD. Additionally, Dong et al [35] showed that disease burden worsened self-management. These findings suggest that healthcare providers should enhance health education for patients with significant anxiety levels, informing them about the meaning and appropriate strategies of self-management to further promote self-management behaviors.

In addition, self-management in patients with anxiety disorders was positively correlated with quality of life, suggesting that better self-management can improve quality of life. Similarly, Yang et al [36] found a positive association between self-management and quality of life. Zhu et al [37] demonstrated that effective self-management interventions for patients with schizophrenia can significantly improve quality of life. These findings suggest the need for healthcare professionals to enhance patient self-management to improve the quality of life of patients with anxiety.

The results of the mediated effect pathway analysis showed that self-management mediated the effect between anxiety and quality of life with an effect value of –0.27, accounting for 38% of the total effect. In another study on hypertension, Qiu et al [38] found that quality of life in patients with hypertension depended on symptom severity and self-management behaviors, and that self-management moderated the relationship between symptoms and quality of life. This finding corroborates the relationship between self-management and anxiety levels and quality of life in patients with anxiety disorders. Additionally, it suggests that further studies should focus on self-management of patients with anxiety disorders after discharge to further improve the quality of life of patients at home.

## Limitation

Our study had some limitations. First, the study participants were patients followed up in a single anxiety disorder outpatient clinic in a large tertiary care hospital. Therefore, future surveys should be conducted at multiple centers with large sample sizes. Second, the study participants included only patients who were literate and could use electronic devices, such as mobile phones. Patients with anxiety disorders living in remote, medically and educationally disadvantaged areas were not included.

## Conclusion

In this study, Self-management of patients with anxiety disorders had a negative correlation with anxiety and positive correlation with quality of life. Additionally, it played a partially mediating effect between anxiety and quality of life. To improve the quality of life of patients with anxiety disorders, much attention should be paid not only to treating disease severity but also to improving self-management practice and ability of patients at home.

## Suggestions

Currently, several studies have assessed self-management interventions for patients with anxiety disorders [33–35]. In addition, some researchers have explored self-management models for patients with anxiety disorders [35]. However, there is not yet an established model of home self-management for patients with anxiety disorders and it is suggested that future research could start from there to improve the quality of life of patients treated at home.

## Supporting information

S1 File (SAV)
